# Myelin Oligodendrocyte Glycoprotein (MOG) Antibody-Associated Disease Presenting as Acute Disseminated Encephalomyelitis Following COVID-19 mRNA Vaccination

**DOI:** 10.7759/cureus.114793

**Published:** 2026-08-19

**Authors:** Monica He, Christopher Bondoc, Robert Fekete

**Affiliations:** 1 Psychiatry, UConn Health, Farmington, USA; 2 Neurology, Brigham and Women's Hospital, Boston, USA; 3 Neurology, New York Medical College, Valhalla, USA

**Keywords:** adem, covid-19, demyelination, myelin oligodendrocyte glycoprotein, vaccination

## Abstract

Acute disseminated encephalomyelitis (ADEM) is a rapidly progressive autoimmune demyelinating condition that primarily affects the central nervous system. The exact pathogenesis is not well understood, but current literature suggests roles for both environmental triggers and genetic predispositions. Myelin oligodendrocyte glycoprotein antibody-associated disease (MOGAD) is an increasingly recognized autoimmune demyelinating disorder that can present with an ADEM-like phenotype, particularly in adults.

We report the case of a 53-year-old Asian-American female patient of Taiwanese ancestry with a past medical history of obstructive sleep apnea and no prior autoimmune disease or family history of demyelinating disorders who presented to the emergency room (ER) with left-sided numbness and weakness, ataxia, vertigo, and slurred speech in the context of a three-month long history of headaches that started one day after she received her first dose of Pfizer COVID-19 vaccine. Brain imaging studies showed findings most suggestive of ADEM. Initial pre-treatment cerebrospinal fluid (CSF) analysis showed markedly elevated protein (473 mg/dL, reference 15-45 mg/dL) with a normal glucose level and mild pleocytosis (6 WBC/µL). Extensive workup was negative for infectious, oncologic, and autoimmune etiologies including negative serum and CSF anti-MOG antibody testing during the acute presentation. The initial treatment of a five-day intravenous methylprednisolone course (250 mg every six hours, total 1,000 mg/day) did not result in significant clinical improvement. The patient was then started on plasma exchange therapy and successfully recovered by the end of the last session with objective improvement in ambulation, dysarthria, and limb strength. The patient tested positive for anti-myelin oligodendrocyte glycoprotein antibody in serum six months after hospital discharge at a titer of 1:32 by live cell-based assay.

This case provides an example of a rare but serious event temporally associated with the SARS-CoV-19 mRNA vaccine and MOGAD presenting as ADEM in a previously healthy adult. The absolute risk of demyelinating events following COVID-19 vaccination is extremely low, and the benefit of vaccination vastly outweighs these rare risks.

## Introduction

Acute disseminated encephalomyelitis (ADEM) is a rapidly progressive autoimmune demyelinating condition that primarily affects the central nervous system. The exact pathogenesis is not well understood, but current literature suggests roles for both environmental triggers such as vaccines and viral infections as well as bacterial infections ​in addition to genetic predispositions. ADEM most commonly presents in children aged 5-8 years old and has a worldwide incidence of 0.3-0.6 per 100,000 per year [1]. Although ADEM most commonly presents in children as fever, meningitis-like symptoms, and loss of consciousness, adult-onset ADEM is primarily characterized by motor and sensory deficits, brainstem dysfunction, and ataxia [2]. The clinical course manifests in the setting of post-viral (for example, influenza, Epstein-Barr virus, cytomegalovirus) or bacterial infection, including *Mycoplasma pneumoniae,* in 50-75% of cases [3]. However, the association between ADEM and vaccines including measles, mumps, rubella, influenza, and rabies has been well documented [4]. Genetic factors for increased risk of ADEM include human leukocyte antigen DRB1*1501 and polymorphisms in TNF-alpha and Interleukin-17 pathways [5]. In the context of the recent COVID-19 pandemic, proper patient education and understanding of the risks and benefits of vaccinations are especially critical. Differential diagnosis in this case included myelin oligodendrocyte glycoprotein antibody-associated disease (MOGAD), multiple sclerosis (MS), autoimmune encephalitis, paraneoplastic encephalitis, viral encephalitis, and Behçet syndrome.

MOGAD is an autoimmune demyelinating disorder distinct from MS and neuromyelitis optica spectrum disorder. MOGAD can present with an ADEM-like phenotype in approximately 3% of adult cases [6]. Unlike classic post-infectious ADEM, MOGAD can follow a relapsing course, and anti-myelin oligodendrocyte glycoprotein antibody testing may be negative in the acute phase with seroconversion occurring weeks to months later. Oligoclonal bands are typically absent in MOGAD, consistent with the findings in this case [7]. 

This case was previously presented as an abstract at the 2022 American Academy of Neurology Annual Meeting [8].

## Case presentation

A 53-year-old Asian-American woman of Taiwanese ancestry presented to the ER of our tertiary trauma center with new-onset left-sided numbness, weakness of the proximal left lower extremity, ataxia, dizziness, and slurred speech. Upon arrival, the patient’s fiancé stated that she had not seemed her usual self and that she had been slow to respond to questions.

**Figure 1:**
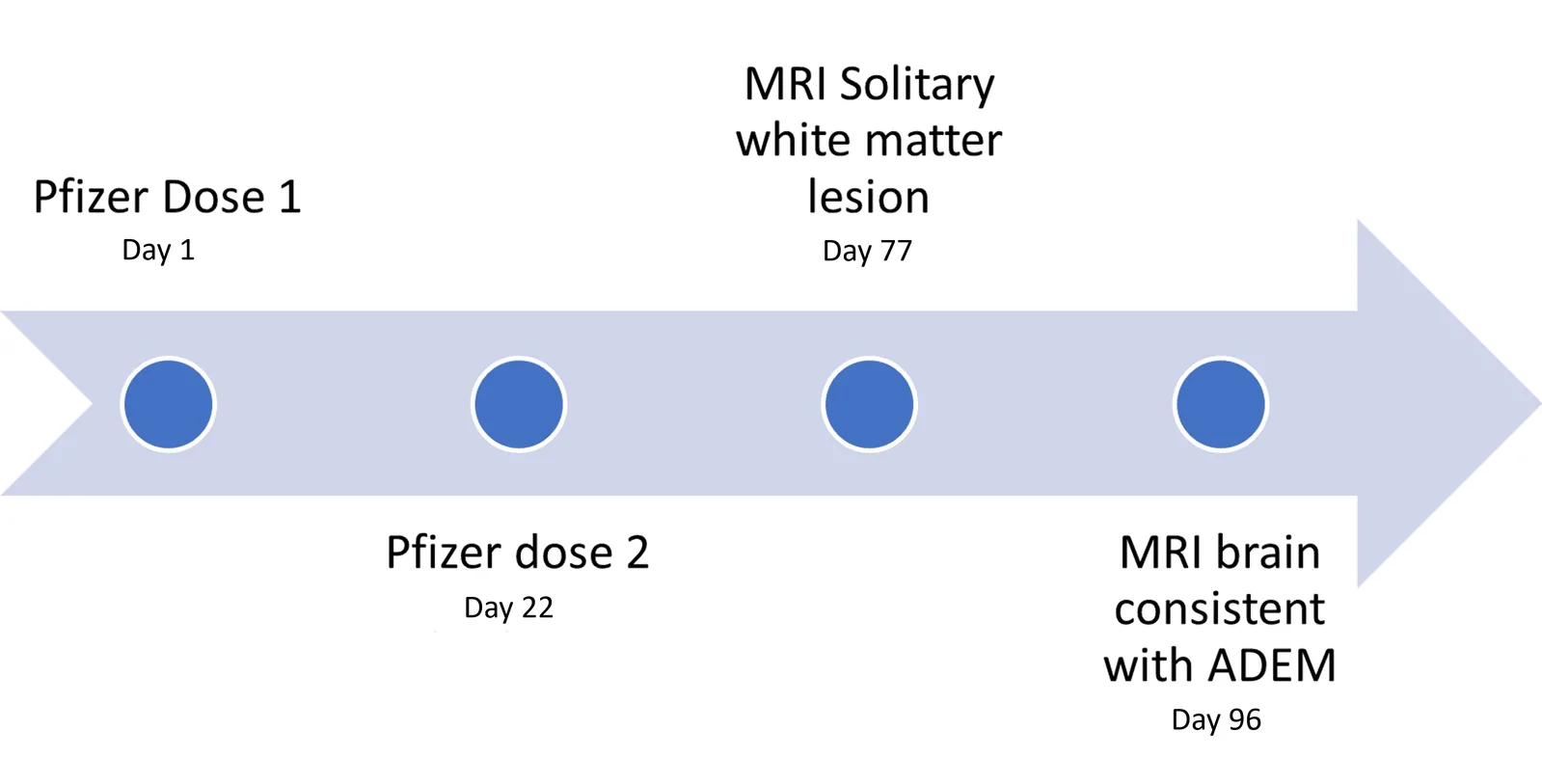
Timeline of initial clinical events The figure was created using Microsoft PowerPoint and Microsoft Paint software. ADEM: Acute disseminated encephalomyelitis

She reported initially developing a headache one day after her first Pfizer COVID-19 vaccine shot three months ago, which was treated with ibuprofen without relief, and worsened such that she had difficulty concentrating (Figure 1).

**Figure 2:**
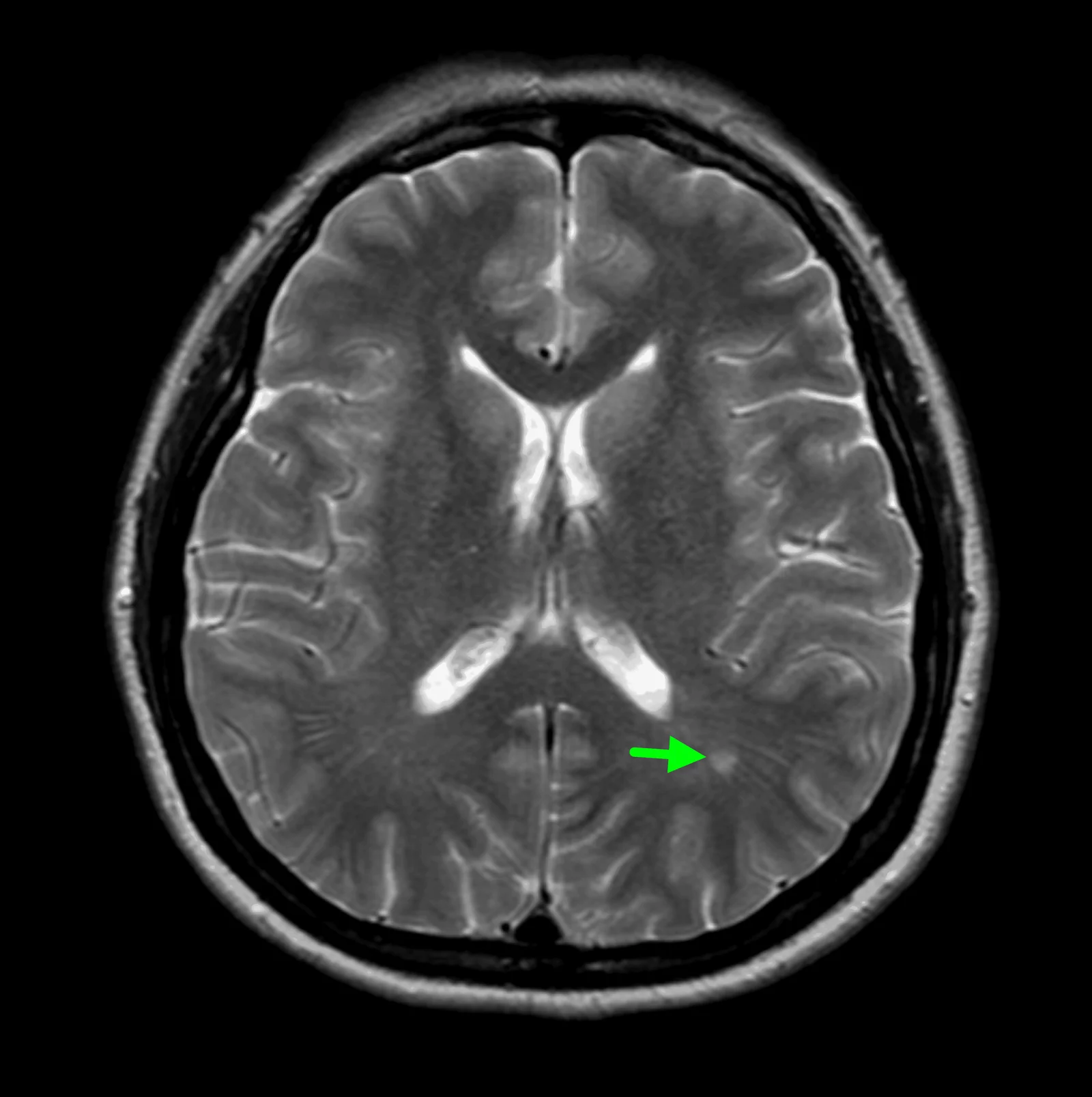
Outside hospital T2 MRI brain showing a single small left-sided white matter lesion (day 77 post initial COVID-19 vaccination) This solitary lesion was initially non-specific but, in retrospect, may represent early preclinical demyelination.

This eventually subsided but she continued to have intermittent headaches coupled with dizziness over the next month. Her symptoms worsened to intractable nausea and vomiting, vertigo, and confusion two weeks after her second vaccine shot, which prompted her to go to another institution’s ER where she was given meclizine and referred to an outpatient neurology service. She was subsequently seen by neurology and had extensive bloodwork including for tick-borne disease and numerous imaging studies including magnetic resonance imaging (MRI), magnetic resonance angiography, magnetic resonance venography, computed tomography (CT) of the head without contrast, and ultrasound of the temporal artery. No formal diagnosis was given at the time, as all workup was reported as negative. Figure 2 of the initial MRI brain on day 77 after the initial COVID-19 vaccination shows a single small left-sided white matter lesion which was not identified at the time as clinically significant.

The patient then developed left-sided facial numbness seven weeks after her initial ER visit, which then progressed into lower left extremity weakness. She was then admitted to the other hospital for overnight treatment with repeated negative stroke workup, which consisted of CT head and carotid Doppler testing, which was reported as negative. MRI brain was not performed at this time. She was discharged on gabapentin. She was experiencing persistent balance difficulty and dragged the left leg during ambulation.

On initial evaluation at our hospital on day 96 after initial COVID-19 vaccination, she was noted to have ataxic gait, decreased left-sided upper extremity sensation, hypometric horizontal saccades, and mild bilateral finger-to-nose dysmetria. No memory, attention, or cranial nerve deficits were found. Reflexes were normal throughout. The patient was then admitted to rule out neurologic versus metabolic etiologies of her symptoms. Initial lab values did not show leukocytosis. Tumor markers and sedimentation rate were within the reference range. Workup for tuberculosis, porphyria, and viral infections was negative. Autoimmune serum workup was also negative (Table 1). 

**Table 1:** Serum and lumbar puncture laboratory investigations Results of serum and lumbar puncture laboratory investigations.

Serum Autoimmune Antibody Investigations	Result	Reference Range
Anti-myelin oligodendrocyte glycoprotein	Negative	Negative
Anti-nuclear	Negative	Negative
Anti-mitochondrial	Negative	Negative
Anti-smooth muscle	Negative	Negative
Anti-cardiolipin	Negative	Negative
Anti-liver kidney microsomal	Negative	Negative
Anti-beta 2 glycoprotein 1	Negative	Negative
Anti-neutrophil cytoplasmic antibodies (ANCA)	Negative	Negative
Angiotensin-converting enzyme (ACE)	32 U/L	16-85 U/L
Sedimentation rate	8	0-30
Lumbar Puncture Laboratory Investigations	Result	Reference Range
Anti-myelin oligodendrocyte glycoprotein	Negative	Negative
Neuromyelitis optica antibody (aquaporin-4)	Negative	Negative
Oligoclonal bands	Negative	Negative
Glucose	65 mg/dL	45-80 mg/dL
Protein	473 mg/dL	15-45 mg/dL
White blood cells	6 cells/µL	0-5 cells/µL
Red blood cells	2 cells/µL	0 cells/µL

**Figure 3:**
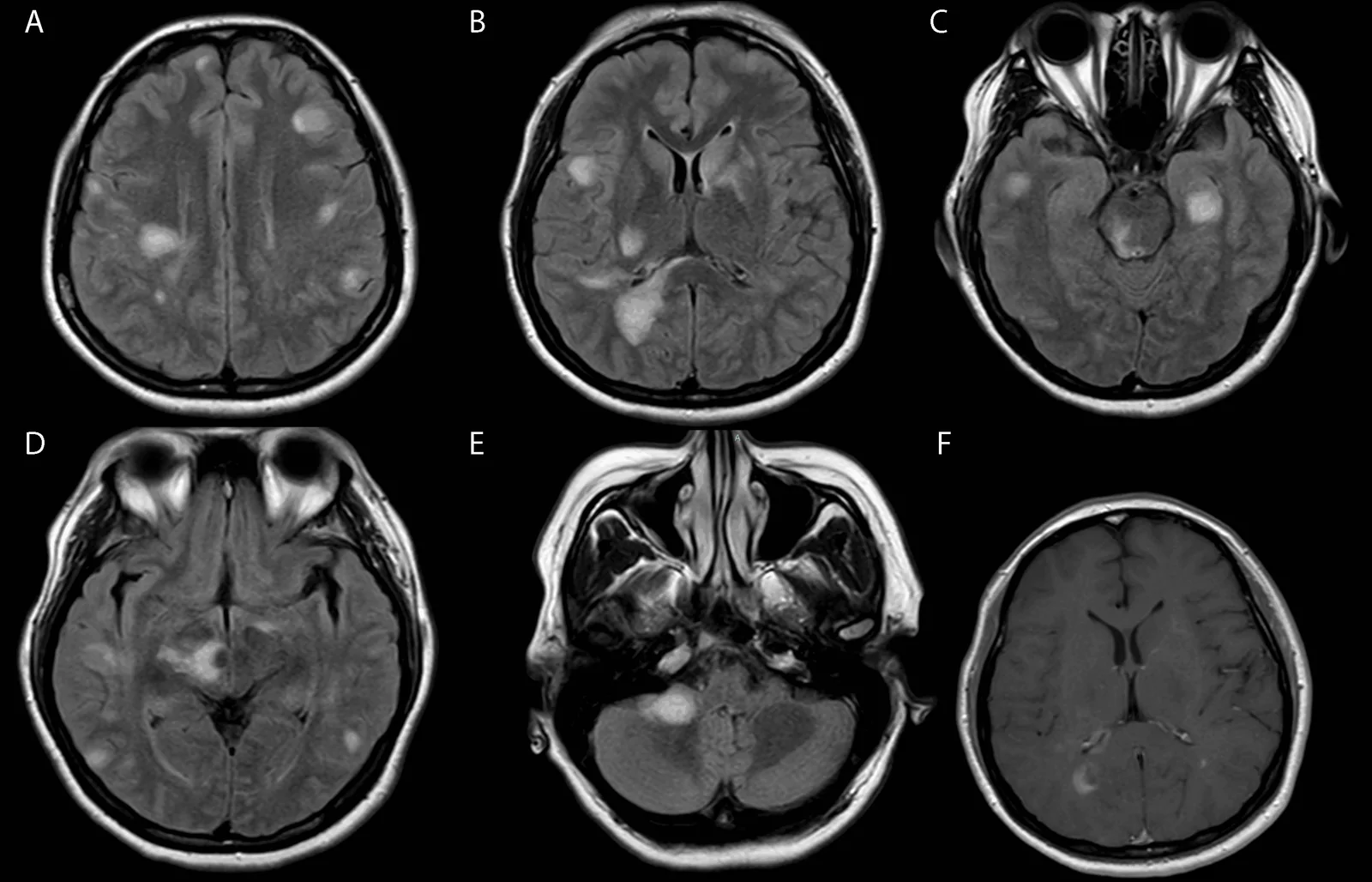
Panel showing white matter lesions on MRI brain T2 FLAIR MRI brain images showing white matter lesions consistent with demyelination, which are subcortical (A and B), subcortical and pontine (C), midbrain and subcortical (D), and cerebellar (E). Enhancement of the parietal lobe lesion is seen in the T1 postcontrast image (F). These large and poorly marginated lesions involving subcortical white matter, brainstem, and cerebellum are characteristic of an ADEM-like/MOGAD phenotype and distinct from the ovoid periventricular lesions typical of MS. Images were obtained on day 96 after initial COVID-19 vaccination. ADEM: Acute disseminated encephalomyelitis; MOGAD: myelin oligodendrocyte glycoprotein antibody-associated disease

The angiotensin-converting enzyme (ACE) level was normal at 32 U/L (normal 16-85 U/L). Anti-neutrophil cytoplasmic antibodies (ANCA) were negative. Initial lumbar puncture (LP) analysis showed remarkably elevated protein levels (473 mg/dL, reference 15-45 mg/dL), normal glucose, and elevated WBC count (6 cells/μL) (Table 1). She had CSF as well as serum anti-Aquaporin-4 (AQP4) antibody and cell-based assay anti-myelin oligodendrocyte glycoprotein (MOG) antibody analysis, which were negative during this acute presentation. Oligoclonal bands were negative. Due to clotting of the initial cerebrospinal fluid (CSF), a repeat LP five days later was taken and was unsuspicious for infection. Both LPs were negative for bacterial, fungal, or viral infections, including SARS-CoV-2 as determined by reverse transcription real-time PCR. CT of the head showed multiple cerebral hypodensities in bilateral frontal and parietal lobes with a question of right occipital lobe involvement near the midbrain. CT angiography (CTA) and CT venography were both unremarkable. MRI of the brain with and without contrast revealed multiple hyperintense lesions involving primarily white matter in the cerebral hemispheres suggestive of demyelination (Figure 3). The lesions were large and poorly marginated, involving subcortical white matter, pons, midbrain, and cerebellum, with incomplete ring enhancement of the parietal lobe lesion. These findings are characteristic of ADEM/MOGAD and distinct from the ovoid periventricular "Dawson's fingers" typical of MS.

MRI of the cervical and thoracic spine with and without contrast both showed no abnormalities. CT of abdomen and pelvis with contrast found a right ovarian cyst. She was started on a five-day course of intravenous (IV) methylprednisolone 250 mg every six hours, for a total daily dose of 1,000 mg for the abnormal MRI findings. This regimen was utilized per institutional protocol for acute demyelinating syndromes at the time of presentation, though we acknowledge that standard guidelines typically recommend 500 mg to 1 g daily as a single infusion. Complications from IV steroids included elevated white blood cell (WBC) count with peak at 35.08 k/mm^3^ as well as hepatic steatosis and transaminitis with aspartate aminotransferase peaking at 86 U/L and alanine aminotransferase peaking at 292 U/L. Ceruloplasmin levels were found to be low at 0.8 mg/dL. Following the completion of IV steroids, she was started on a one-week course of 60 mg prednisone PO with a taper in the subsequent weeks.

Due to her continued difficulty walking, unresolved left-sided weakness, and development of waxing and waning mental status after receiving IV steroids, she was started on five sessions of plasma exchange therapy (PLEX). Prior to PLEX, the patient was unable to ambulate independently, had persistent dysarthria, and left lower extremity weakness rated as Medical Research Council (MRC) grade 3/5. Following five sessions of PLEX, she demonstrated independent ambulation with cane, resolution of dysarthria, and improved strength to MRC grade 4+/5, supporting PLEX efficacy in this steroid refractory case. The patient ultimately improved on PLEX and was discharged following her fifth and final session of plasmapheresis with plans to follow up with outpatient neurology.

**Figure 4:**
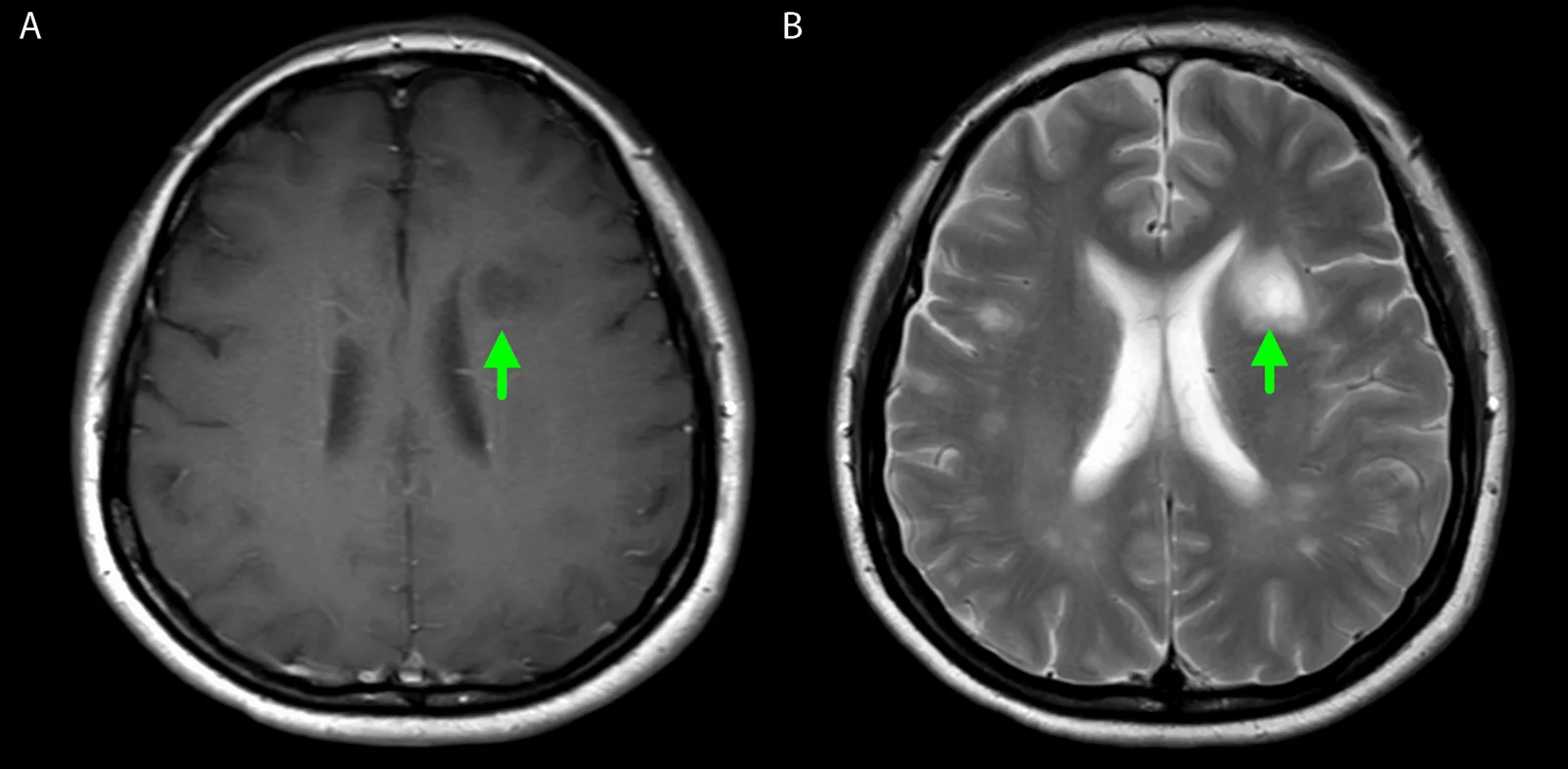
Repeat MRI brain on day 203 after initial COVID-19 vaccination post-contrast T1 (A), non-contrast T2 (B), demonstrating a new non-enhancing T2 hyperintense lesion involving the left corona radiata/centrum semiovale (arrows), suspicious for a new demyelinating lesion, as well as interval decrease in conspicuity of the numerous, previously demonstrated parenchymal lesions, with resolution of associated contrast enhancement. The new lesion suggests a relapsing MOGAD phenotype rather than monophasic ADEM. Repeat MRI brain on day 203 after initial COVID-19 vaccination post-contrast T1 (A), non-contrast T2 (B), demonstrating a new non-enhancing T2 hyperintense lesion involving the left corona radiata/centrum semiovale (arrows), suspicious for a new demyelinating lesion, as well as interval decrease in conspicuity of the numerous, previously demonstrated parenchymal lesions, with resolution of associated contrast enhancement. The new lesion suggests a relapsing MOGAD phenotype rather than monophasic ADEM. ADEM: Acute disseminated encephalomyelitis; MOGAD: myelin oligodendrocyte glycoprotein antibody-associated disease

On follow-up 65 days after discharge, the patient reported complete resolution of her balance problem, gradual progress in recovery of her baseline physical strength, continued difficulty with concentration requiring effort with only modest improvements, and persistent numbness and tingling in her left hand and face. Repeat MRI brain on day 203 after initial COVID-19 vaccination showed a new non-enhancing T2 hyperintense lesion involving the left corona radiata/centrum semiovale, suspicious for a new demyelinating lesion, as well as interval decrease in conspicuity of the numerous, previously demonstrated parenchymal lesions, with resolution of associated contrast enhancement (Figure 4). The appearance of a new asymptomatic lesion more than three months after the index event challenges a monophasic ADEM diagnosis and raises concern for a relapsing MOGAD phenotype.

Six months after discharge, repeat serum anti-MOG antibody was found to be positive at 1:32 (normal <1:32) by live cell-based assay. At this time, the patient was independently ambulatory which yielded a modified Rankin scale score of 3. 

## Discussion

At the time of publishing this report, there have been only a small handful of primarily case reports within the current literature detailing the relationship between COVID-19 vaccinations and ADEM. Thus far, there have been only two reported cases of ADEM, each following mRNA and inactivated SARS-CoV-2 vaccinations. Vogrig et al. reported on a female patient with a past medical history significant for postinfectious rhombencephalitis who presented with subacute hemiataxia [9], which improved after a course of methylprednisolone. Kania et al. described a young healthy female patient who presented with fever, meningitis-like symptoms, and urinary retention [10], which also resolved on steroids. The two patients who developed ADEM following vaccination with the inactivated vaccine were female and had received the Sinovac vaccine within one month of the clinical onset of symptoms [11,12]. One of the patients had no significant past medical history and presented with fever, headache, and fatigue, a set of symptoms that manifest more commonly in younger patients; this patient was successfully treated with methylprednisolone and intravenous immunoglobulin infusions [12,13]. The other patient had a history of Hashimoto’s thyroiditis and presented to the hospital following an episode of tonic-clonic seizure; clinical and radiographic improvement occurred following a course of steroids as well [11]. We discuss a patient with no previous significant medical history whose clinical course was refractory to methylprednisolone and only started to resolve after multiple sessions of plasmapheresis.

Due to the rapidly progressive nature of the patient’s clinical course, the differential diagnosis was focused on acute neurologic disorders. We systematically considered MS, autoimmune encephalitis, paraneoplastic encephalitis, infectious etiologies, neuromyelitis optica spectrum disorder (NMOSD), Behçet syndrome, sarcoidosis, porphyria, and CNS vasculitis. 

The subacute onset of a single episode of diffuse demyelination, in addition to the lack of finding of oligoclonal bands in CSF and the lack of characteristic MS symptoms such as visual abnormalities, bladder and bowel dysfunction, and fatigue, does not favor a diagnosis of MS. Additionally, MRI lesions were large and confluent rather than the small ovoid periventricular lesions typical of MS.

The lack of hallmark symptoms, such as psychosis, seizure, memory deficit, and dyskinesia, suggests against autoimmune encephalitis.

Normal tumor markers, absence of oligoclonal bands in CSF, and lack of radiographic findings suspicious for common associated neoplasms such as lung and breast cancer altogether are not consistent with paraneoplastic encephalitis. Negative bacterial, viral, and fungal titers in CSF rule out infectious etiologies. Negative AQP4-IgG in serum and CSF, absence of optic neuritis, and absence of longitudinally extensive transverse myelitis argue against NMOSD.

Although neurologic manifestations can occur in 9% of patients [14], Behçet syndrome typically presents with oral or genital aphthous ulcers rather than in the absence of these symptoms [15]. Eosinophilic CNS vasculitis can mimic the imaging appearance of demyelinating disease [16]. Vasculitis due to eosinophilic granulomatosis with polyangiitis (Churg-Strauss syndrome) was thought to be less likely given negative ANCA testing and absence of systemic manifestations. Similarly, there were no signs of systemic sarcoidosis such as mucosal ulcerations and ACE level was normal. CT angiography of the head and neck was normal. Biopsy to rule out isolated eosinophilic CNS vasculitis or primary CNS vasculitis was not performed, which is a limitation of the workup. Urine porphobilinogen, delta-aminolevulinic acid, and total porphyrins were within the normal range, which rules out acute intermittent porphyria (AIP). AIP is a rare cause of ADEM. 

Initially, MOGAD was considered less likely given negative acute-phase serum and CSF MOG-IgG. However, MOG-IgG seroconversion at six months (titer 1:32 by live cell-based assay), combined with the ADEM-like phenotype, absence of oligoclonal bands (positive in <20% of MOGAD), and the new lesion on day 203 MRI, supports a final diagnosis of MOGAD presenting with an ADEM-like phenotype [17].

Considering the clinical course as well as both radiologic and CSF findings of pleocytosis and absence of oligoclonal bands, a diagnosis of ADEM was given. Only 3% of adult anti-MOGAD patients present with ADEM [6]. Initial false-negative anti-MOG antibody testing may occur in this disorder. False-negative MOG-IgG in the acute phase is well-recognized, particularly when testing is performed early in the disease course or if assays lack optimal sensitivity. The live cell-based assay at follow-up has higher sensitivity than fixed assays or ELISA [18]. The low positive titer of 1:32, while at the threshold of positivity, is clinically meaningful in the context of a compatible phenotype and negative AQP4-IgG. However, we acknowledge that low titers can occasionally represent transient non-pathogenic antibodies and repeat testing to confirm persistence is recommended. In retrospect, negative oligoclonal bands are consistent with MOGAD, as oligoclonal bands are positive in less than 20 percent of MOGAD patients [7].

The remarkably elevated CSF protein (473 mg/dL) in this case is atypical for classic ADEM, where protein is usually mildly elevated (50-100 mg/dL). Such marked elevation can be seen in severe MOGAD with extensive blood-brain barrier disruption, but can also raise concern for infectious polyneuropathies or spinal block. In this case, the elevated protein likely reflects severe neuroinflammation and extensive barrier breakdown consistent with a fulminant demyelinating process, particularly given the negative infectious workup and compatible clinical-radiological phenotype. 

While current literature has seen a steady increase in reported cases of the association between COVID-19 infection and ADEM, in contrast, the relationship between COVID-19 vaccination and ADEM still requires careful scientific examination and discussion. We emphasize that this single case report describes a temporal association and cannot establish causation. Per the Bradford Hill criteria causality cannot be inferred from an n=1 observation. As with many other vaccines, the majority of patients do not exhibit severe adverse effects such as ADEM. Ismail and Salama provide an in-depth analysis of demyelinating events following COVID-19 vaccination [19]. In their review, 5/32 reported cases were ADEM-like. mRNA vaccines were reported as most likely to precede demyelinating episodes compared to viral vector vaccines. Inactivated vaccines were the least likely to be associated with demyelination. Self-adjuvantation exhibited by mRNA vaccines is one proposed theory for their association with demyelinating episodes. The average time interval to symptom development was 14 days from vaccination, which is shorter than in this case. A lethal case of ADEM following the viral vector AstraZeneca COVID-19 vaccine was not included in the above review of 32 cases [20]. In addition to demyelinating events, there is a report of orbital inflammation after COVID-19 vaccination. Gencoglu reports a case of orbital pseudotumor with left blepharoptosis and lacrimal gland enlargement as well as enlargement of the left lateral and superior rectus muscles, starting one week after the first Pfizer-BioNTech mRNA vaccination and treated with corticosteroids [21].

There are individual case reports of ADEM after COVID-19 natural infection listed by Permezel et al. [20]. It is not possible to make a comparison of ADEM risk between vaccination and natural infection based on case reports. Our patient has consistently received negative COVID-19 PCR test results. Ismail and Salama discuss a theoretical risk of starting an autoimmune reaction post mRNA vaccination via activation of Toll-like receptors TLR7 and TLR8, leading to interferon production and subsequent T and B cell response [19]. The absolute risk of ADEM or MOGAD following COVID-19 vaccination is extremely low (estimated at less than 1 per million doses), while the risk of neurological complications from SARS-CoV-2 infection itself such as stroke, encephalopathy, and Guillain-Barré syndrome, is orders of magnitude higher. The benefits of COVID-19 vaccination in preventing severe infection, hospitalization, and death vastly outweigh these rare risks.

## Conclusions

This case provides an example of a rare but serious complication temporally associated with the SARS-CoV-19 mRNA vaccine and MOGAD presenting as ADEM. Given the major impact and societal disruptions of the COVID-19 pandemic throughout the world, inquiries into the neurologic pathophysiology of vaccine-associated side effects are warranted and should be prioritized. This case was subsequently demonstrated to have serum MOG positivity. Clinicians should keep the possibility of MOGAD in the differential diagnosis of ADEM-like presentations. Negative oligoclonal bands were also consistent with MOGAD. Long-term follow-up and maintenance immunotherapy should be considered given the risk of relapse in MOGAD.
